# The Characteristics of Vascular Growth in VX2 Tumor Measured by MRI and Micro-CT

**DOI:** 10.1155/2012/362096

**Published:** 2011-09-15

**Authors:** X.-L. Qi, J. Liu, P. N. Burns, G. A. Wright

**Affiliations:** Imaging Research, Sunnybrook HSC, University of Toronto, 2075 Bayview Avenue, Toronto, ON, Canada M4N 3M5

## Abstract

Blood supply is crucial for rapid growth of a malignant tumor; medical imaging can play an important role in evaluating the vascular characterstics of tumors. Magnetic resonance imaging (MRI) and micro-computed tomography (CT) are able to detect tumors and measure blood volumes of microcirculation in tissue. In this study, we used MR imaging and micro-CT to assess the microcirculation in a VX2 tumor model in rabbits. MRI characterization was performed using the intravascular contrast agent Clariscan (NC100150-Injection); micro-CT with Microfil was used to directly depict blood vessels with diameters as low as 17 um in tissue. Relative blood volume fraction (rBVF) in the tumor rim and blood vessel density (rBVD) over the whole tumor was calculated using the two imaging methods. Our study indicates that rBVF is negatively related to the volume of the tumor measured by ultrasound (*R* = 0.90). rBVF in the tissue of a VX2 tumor measured by MRI *in vivo* was qualitatively consistent with the rBVD demonstrated by micro-CT *in vitro* (*R* = 0.97). The good correlation between the two methods indicates that MRI studies are potentially valuable for assessing characteristics or tumor vascularity and for assessing response to therapy noninvasively.

## 1. Introduction

Medical imaging is an important and useful tool for assessing the shape and structure of a tumor as it grows and for monitoring the effects of clinical treatments [1–3]. It has been documented that increased vascularity in the neoplastic tissue provides a valuable indication of tumor aggressiveness; this blood supply is crucial for the malignant tumor to grow rapidly. Traditionally, the response to cancer treatment is judged by the reduction in tumor volume. Recently, switching the target of cancer treatment from the exceptionally heterogeneous tumor cell population to the considerably more homogeneous tumor vasculature has emerged as a revolutionary therapeutic approach. Development of a large number of antivascular and antiangiogenic therapies has created the need for techniques that noninvasively quantify vascular volume and flow changes in response to the therapy. Assessment of structural and functional abnormalities of a tumor's blood vessels for prognostic reasons, therapy monitoring, or prediction of therapy success is increasingly gaining attention. The relative blood volume fraction in selected regions (rBVF: a ratio of average signal contribution in a region of the tumor due to intravascular agents relative to that in whole blood, measured *in vivo*), can be determined by 2D MRI using intravascular contrast agents [4, 5]. The relative blood vessel density in the tumor (rBVD: percentage of pixels within identifiable blood vessels relative to total number of voxels in the tumor or tumor region) is best measured by high-resolution micro-CT *in vitro* but may be approximated by high-resolution 3D MRI *in vivo*.

A rabbit model with VX2 tumor has been studied widely to assess tumor development and treatment response [6–8]. In general, the tumor mass can be visualized as two distinct regions: the highly vascular advancing rim where tortuous blood vessels are abundant in the peripheral area and the hypovascular central region where the number of blood vessels gradually decreases, resulting in hypoxia and necrosis. Tumors usually develop a disorganized microvascular network during growth; it has been shown that the density of the blood vessels in the tumor changes at different stages of the tumor growth [9]. The rBVF and rBVD and the changes of blood volume and vascular density with tumor growth can be monitored using medical imaging. However, the relationships between the functional rBVF measured by 2D MRI *in vivo*, morphological rBVD measured by micro-CT *in vitro*, and the size (volume) of the tumor have not yet been reported. Therefore, the purpose of this study was to evaluate the rBVF characteristics in the highly vascular advancing rim of a rabbit VX2 tumor model by 2D MRI *in vivo* and compared to rBVD measured by micro-CT *in vitro* to validate a practical *in vivo* MRI method which could be used to characterize the earlier functional response of a tumor to clinical intervention and treatments. The blood vessel density (rBVD) in the tumor demonstrated by micro-CT and rBVD by 3-dimensional (3-D) MRI were also compared. The relationships among rBVF and rBVD and the volume of the VX2 tumor as it grows were evaluated as well. Micro-CT can demonstrate the tumor's 3D microangioarchitecture and therefore can serve as a gold standard for perfused blood volume; the minimal diameter of the blood vessels detectable on micro-CT images was about 9 to 17 *μ*m [10, 11]. Histopathology was also performed to demonstrate differences in the structure of blood vessels in small and large tumors. 

## 2. Materials and Methods

### 2.1. Animal Model

Nine New Zealand white rabbits (4 to 4.5 kg) were used in this study. All animals were prepared in accordance with the “*Guide to the Care and use of Experimental Animals* (Canadian Council on Animal Care)” under approved protocols. The rabbits were each injected intramuscularly with about 1 × 10^6^ VX2 tumor cells in a hind leg. The tumor was monitored daily by B-mode ultrasound starting at 7 days after injection of the tumor cells. When the tumor reached at least 2 cm^3^, an MRI study was scheduled. Tumor volume was calculated at the time of the MRI study from the maximum length, width, and height measured on ultrasound images acquired the same day as length (cm) ∗ width (cm) ∗ height (cm) ∗  *π*/6, assuming a prolate ellipsoid shape. Tumors were included with volumes ranging from 2.4 cm^3^ to 15.37 cm^3^ based on studies at time points ranging from 10 to 20 days after the tumor cells were injected. MRI and micro-CT studies, for each tumor were conducted within 24 hours. Rabbits were anaesthetized with Ketamine (50 mg/kg) and Xylazine (5 mg/kg).

### 2.2. MRI

Studies were performed on a GE Signa 1.5 T CV/i equipped with 40 mT/m gradients with a 150 mT/m/s slew rate using a 3-inch receive-only surface coil for signal reception. The maximal center cross-section of the tumor were imaged by 2D MRI with a *T*_1_-measurement acquisition.

#### 2.2.1. 2D MRI

2D Quantitative *T*_1_ measurements for rBVF were made using a Look-Looker sequence with spiral readouts (225 ms acquisition spacing, TR = 3000 ms, RBW = 125 kHz, 20° flip angle, 7 mm slice thickness, FOV = 20 cm, 4096 × 8 spiral readouts), 1.1 mm resolution. The intravascular contrast agent, Clariscan, (NC100150-Injection, GE Healthcare) was used in this study. *T*_1_ measurements were made both prior to and approximately 4 minutes following an intravenous bolus injection of Clariscan (0.05 mL/kg = 0.15 mg Fe/kg), and then at 15-minute intervals of different time points thereafter. Dynamic *T*_1_-weighted imaging for perfusion was performed immediately following Clariscan injection and used for identifying the region of interest (ROI) in the tumor rim (fast spoiled gradient echo 2DFT sequence, TR = 12 ms, TE = 5 ms, 30° flip angle, bandwidth = 15.63 kHz, Matrix = 256 × 128, FOV = 20 cm). 

A total of 10 mL (5 × 2.0 mL tubes) of arterial blood was drawn both before and after the Clariscan injection at different time points and *T*_1_ decay times for each sample was measured (TR = 3000 ms, TE = 50 ms, RBW = 125 kHz, 10° flip angle, 10 mm slice thickness, FOV = 20 cm, 6 NEX, 4096 × 4 spiral readouts) to calculate the *T*_1_ due to the contrast agent in whole blood. The changes in longitudinal relaxation rates *R*_1_ (1/*T*_1_) in blood after administration of contrast medium is proportional to contrast agent concentration. Under the assumption that Clariscan remains intravascular in the tumor, the blood volume fraction can be calculated using the equation:

(1) $$\text{BVF}=\frac{\left(1/T_{1}t\right)\text{postCA}-\left(1/T_{1}t\right)\text{preCA}}{\left(1/T_{1}b\right)\text{postCA}-\left(1/T_{1}b\right)\text{preCA}},$$

where preCA and postCA refers to measures before and after injection of Clariscan. *T*_1_ *t* and *T*_1_ *b* refer to the measurements from the tissue *in vivo* and the blood sample *in vitro*, respectively. This equation assumes that we can model the tumor as two pools, consisting of an intravascular and extravascular compartments, and the distribution of protons between the pools is equal (partition coefficient = 1). In addition, the exchange of protons between the pools is fast enough that the system can be regarded as well mixed within a time on the order of *T*_1_ [12]. Previous studies showed that the condition of fast water exchange appears to be met when *T*_1_ in blood is greater than 150 ms [13, 14]. The postCA measurement of Clariscan for rBVF measurement was taken at the last time point (at 60 to 90 min after injection) when *T*_1_ of blood was >150 ms to satisfy this condition.

#### 2.2.2. 3D MRI

The 3D spoiled gradient echo acquisition (3D SPGR, TR = 6 ms, TE = 2 ms, FOV = 20 cm, 30° Flip angle, Bandwidth = 125 kHz, slice thickness = 0.8 mm, 320 × 320 pixels in-plane yielding approximately 0.6-mm resolution) was also conducted before and 10 minutes after Clariscan injection. Maximum Intensity Projection (MIP) was obtained by subtraction of pre- from post-contrast agent.

### 2.3. Micro-CT

When MRI *in vivo* studies was finished, tumors were perfused via the femoral artery with saline followed by microfil (Flow Tech inc., Carver, MA) for the micro-CT study. The tumor was excised 90 minutes after microfil perfusion to allow time for the microfil to harden; the tumors were then stored in 10% formalin for 24 hours. The specimen was then mounted in 10% gelatin and 3D cone beam CT data sets were acquired over 2.5 hours with 905 views at 35 *μ*m-resolution using a micro-CT scanner (MS-8, GE Medical Systems, London, Ontario). An X-ray source of voltage 80 kvp and a beam current 90 *μ*A were used. A 3D data volume was reconstructed using the Feldkamp algorithm for cone beam CT geometry [11]. 3D surface rendering of the vasculature of tumor was accomplished using Display software (Montreal Neurological Institute, McGill, Montreal, Canada).

### 2.4. Tissue Preparation and Histology

Since microfil used for micro-CT makes it difficult to interpret histology, we examined samples from four additional VX2 tumors of similar sizes histologically to confirm vascular characteristics. These were fixed in 4% PFA for 4 hours, washed in PBS 3 times for 15 minutes, and cryoprotected in 15% sucrose in PBS for 1 hour followed by 30% sucrose in PBS overnight at 4°C. Samples were then incubated in Tissue-Tek OCT (Sakura) at 4°C for 4 hours prior to embedding in OCT over dry ice. The tissue blocks were cryosectioned at 7 *μ*m, placed onto L-polylysine-coated slides (Fisher Scientific), and dried for 1–4 hours at room temperature before storage at −20°C. Histology study was conducted by Immunohistochemistry staning, the slides were incubated with alkaline phosphatase conjugated monoclonal anti-*α*-Smooth muscle actin (1 : 100; Sigma) for 1 hour at room temperature followed by washing 4 times in PBS for 5 min. This stain was used to identify microvessels based on their smooth muscle layer and was found to be adequate for this purpose in the rabbit model. Then the sections were incubated 10 min in alkaline phosphatase buffer (100 mM Tris-HCl, pH 9.5, 100 mM NaCl, 10 mM MgCl_2_) and then stained with BM Purple AP substrate (100 mM Tris-HCl, pH 9.5, 100 mM NaCl, 50 mM MgCl_2_, 0.01% sodium deoxycholate, 0.02% NP-40, 337 mg/mL NBT (nitroblue tetrazolium salt; Boehringer Ingelheim), and 175 mg/mL BCIP (5-bromo-4-chloro-3-indolyl phosphate, toluidinium salt; Boehringer Mannheim). The staining reaction was allowed to proceed for 10 min at room temperature. The slides were then washed extensively in PBS and covered with cytoseal mounting medium (Richard-Allan Scientific).

## 3. Data Analysis

### 3.1. 2D MRI

ROIs were carefully placed around the abundant microvascular rim of the tumor in both large and small tumors for 2D measurements. The ROI in the rim of tumor tissue avoids necrosis and bigger vessels in the center of the tumor for correctly assessing the rBVF in microcirculation. The ROIs were determined from serial images taken immediately after injection of the contrast agent Clariscan (published data) [15] in MRI as areas that were filled with contrast agent at early phases and showed clearer outlines for the tumor.

### 3.2. 3D MRI and Micro-CT

Measurements of vessel density, rBVD, from 3D MRI and 3D micro-CT were determined by rendering 3D isosurfaces in Amira (AmiraDev 4.1.1; TGS, Berlin, Germany). Micro-CT images *in vitro* showed the isolated tumor's vessel clearly, however, Images of 3D MRI *in vivo* demonstrated not only the tumors' vessels but also the blood vessels of whole leg, Therefore the MRI images and micro-CT images were aligned using the big blood vessels in the tumor, then the tumor was segmented by manually drawing a volume using “Label/Voxel” to encompass the whole tumor defined by the vascular rim in the micro-CT data. The blood vessel density over the tumor (rBVD) was determined by counting voxels inside the surface-rendered vessels and comparing that to the total number of voxels in the tumor using the “TissueStatistics” package in Amira.

## 4. Statistical Analysis

The correlations between rBVF in the rim measured by 2D MRI and the volume of the tumor by ultrasound were assessed with Pearson's correlation coefficient. The relative blood volume fraction in 2D and the blood vessel density in 3D MRI were compared. The blood vessel density determined by 3D MRI and micro-CT were also compared. Statistical significance was set as *P* < 0.05.

## 5. Results

### 5.1. rBVF Relative to Volume of the Tumor

**Figure 1:**
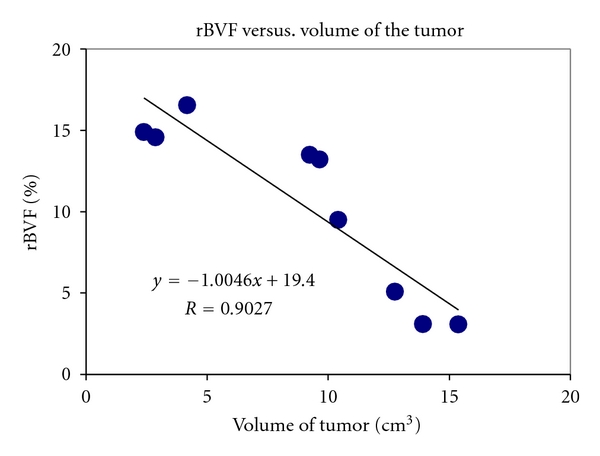
rBVF measured by MRI in a VX2 tumor is inversely related with volume of tumor (cm^3^). *R* = 0.90, *P* < 0.001.

Our results indicated for the first time that rBVF in the rim of the tumor seems to decrease with increasing volume of VX2 tumors (as measured by ultrasound) once the tumors were detectable and measurable for rBVF (i.e., those larger than 2.4 cm^3^ in this study). There was a significant inverse relationship between the rBVF and the volume of the tumor, *R* = 0.90, *P* < 0.001 (Figure 1). In our study, the rBVF in the tumor's rim varied from 3.05 to 16.55% with an average of 10.38 ± 5.36%, as measured by MRI; the volume of the tumors ranged from 2.40 to 15.37 cm^3^ with an average volume of 8.98 ± 4.82 cm^3^, obtained by ultrasound.

### 5.2. rBVF in 2D Compared to rBVD in 3D MRI

**Figure 2:**
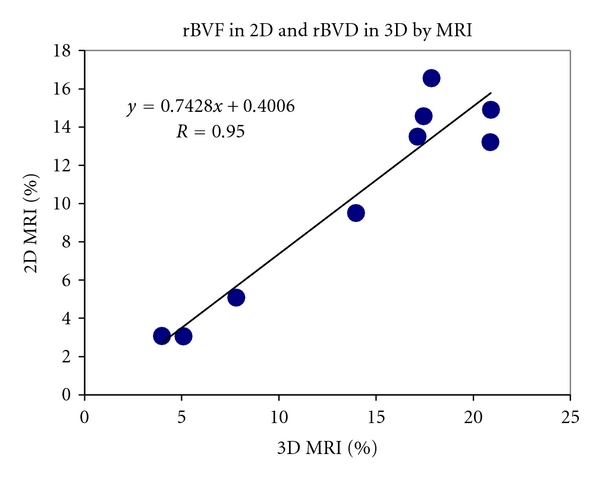
Relationship between rBVF measured by 2D MRI and rBVD by 3D MRI. *R* = 0.95, *P* < 0.001.

The rBVF in the rim measured by 2D MRI was significantly correlated with the rBVD across the tumor calculated from 3D MRI (Figure 2, *R* = 0.95, *P* < 0.001). The rBVF in the rim measured in this study was derived in a slice at the maximum cross-section of the tumor, and rBVD in 3D was calculated from the whole volume of the tumor. The average rBVD in 3D was higher than the rBVF measured in 2D, likely due to the greater coverage of tumor and inclusion of larger vessels in the 3D acquisition.

### 5.3. Blood Vessel Density in Micro-CT Compared to 3D MRI

**Figure 3:**
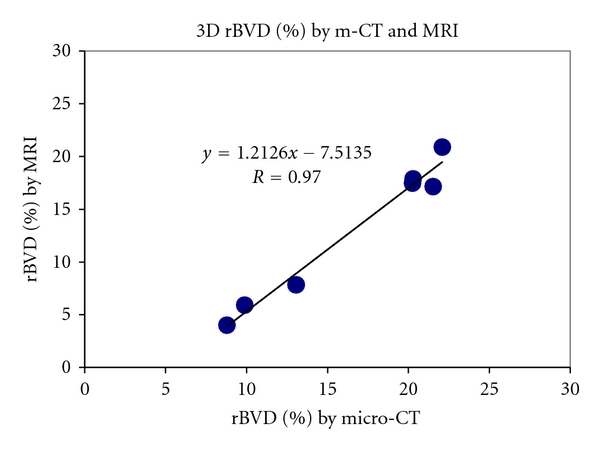
Relationship of blood vessel density measured in 3D by m-CT and MRI. *R* = 0.97, *P* < 0.001.

**Figure 4:**
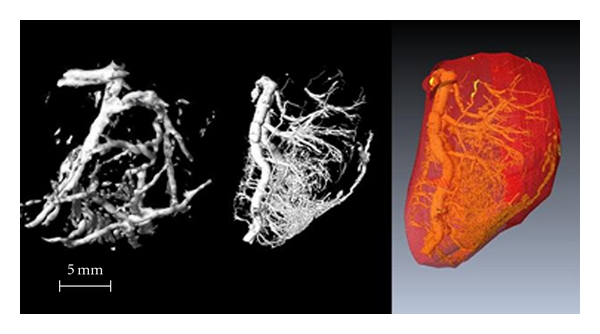
Small tumor (2.8 cm^3^)—left: 3D MRI, middle: micro-CT, right: whole tumor volume. A higher density of blood vessels is seen in the peripheral rim. The large artery on the left side of the images is the feeding blood vessel for the tumor.

**Figure 5:**
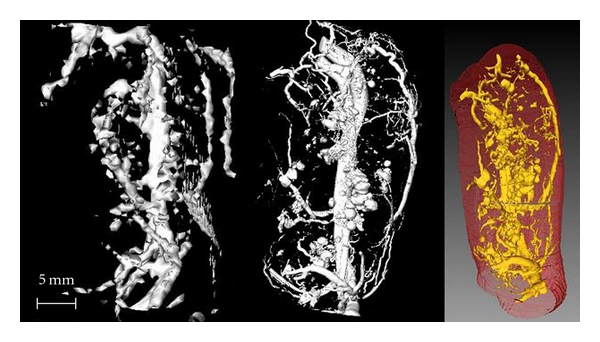
Large tumor (9.6 cm^3^)—left: 3D MRI, middle: micro-CT, right: whole tumor volume. A lower density of blood vessels is seen in the peripheral rim.

The vessel density obtained by micro-CT yielded results similar to corresponding measurements by 3D MRI (Figure 3, *R* = 0.97, *P* < 0.001). A representative large tumor (>9.6 cm^3^ in volume) had a lower density of blood vessels (8.8% in micro-CT compared to the 4.0% derived from MRI). The blood vessel density increased to 20% by micro-CT and to 17% by 3D MRI in a small tumor around 2.8 cm^3^ in volume. The smaller tumor demonstrated a thicker rim with more blood vessels (Figure 4) while the larger tumor showed a thin rim with fewer blood vessels (Figure 5) by 3D MRI and micro-CT.

**Figure 6:**
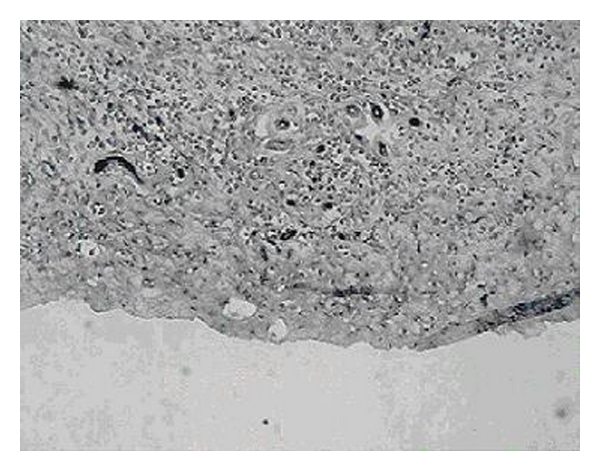
Histology section from a small tumor shows a higher density of smaller vessels.

**Figure 7:**
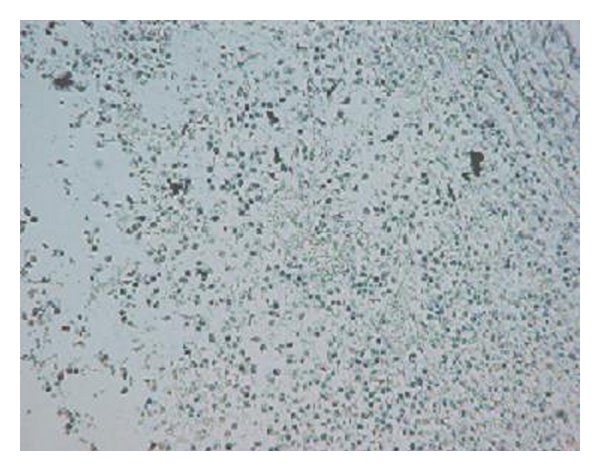
Histology section from a large tumor shows fewer and relatively bigger vessels and necrosis.

The imaging results were consistent with histopathological observations (Figures 6 and 7). In the histology specimens, the small tumor showed more small vessels in the section, while the large tumor showed fewer blood vessels with greater diameters as well as more necrosis.

## 6. Discussion

Our results indicate that the rBVF in the tumor rim measured by noninvasive imaging methods was negatively related to the volume of the tumor and that small tumors at earlier stages of growth have a higher density of blood vessels than in large tumors at the later stages of growth. Therefore, the vasculature of the tumors changed at different growth stages [16, 17]. The higher rBVF and density of blood vessels in small tumors may be related to their rapid growth and the relative absence of necrosis. The large mature tumor has a lower BVF and bigger vessels with more arteriovenous shunts; these characteristics may be related to the greater metastatic potential and the lower efficiency of the treatments at later stages. 

Micro-CT, using a microfocus X-ray source and high-resolution detectors, could clearly observe the 3D microangioarchitecture of the tumor *in vitro*. Interestingly, the images were comparable with 3D MRI at much lower resolution using intravascular contrast agents *in vivo* (Figures 4 and 5). The minimal diameter of the microvessel demonstrated by micro-CT was 17 *μ*m. The resolution in 3D MRI *in vivo* was 0.6-0.7 mm in this study. This suggests that most of the vessel area across the whole tumor is dominated by the larger vessels.

Studies over the last decade have demonstrated that although the microvessel density in tumors is heterogeneous, the vasculature of the tumor may differ substantially at the different stages of the growth [9]. Our results provided imaging and histopathological evidence supporting these conclusions. At the earlier stages of the tumor's growth, more blood vessels were shown in the peripheral rim of the tumor. This could explain the rapid tumor growth and the greater efficiency of treatments in earlier stages.

Clinical investigations have suggested that the incidence of metastases in many cancer types is positively correlated with the microvessel density in vascular hot spots of the primary tumor [18–20]. Metastatic potential also could be related to the increased prevalence of arteriovenous shunts. These may facilitate extravasation and transportation of larger cancer cell clusters. The structural and functional abnormalities of the vessels in the bigger tumors also result in regions that are poorly perfused and hence hypoxic. Some studies have indicated that tumor hypoxia may also promote tissue metastasis. Rofstad has stated in detail the theoretic reasons of several mechanisms involved [21]. Hypoxia is also a cause of resistance to certain medical and cytotoxic agents. The poorly perfused, hypoxic tumor microenvironment may also be resistant to radiation therapy, photodynamic therapy, chemotherapy, and some forms of gene therapy. 

Measurement of microvessel characteristics can aid in assessing the stage of disease, the likelihood of metastasis, and prognosis under various treatment strategies for a wide range of cancers [22–25]. Many studies have demonstrated reductions in blood volume and blood vessel density in tumors after antivascular and anti-angiogenic treatments [26]. However, there are some reports indicating that microvessel density in the tumor is not always an indicator of anti-angiogenic therapeutic efficiency. These data demonstrate that the absence of a drop in microvessel density does not indicate that the agent is ineffective [27–29]. Because anti-angiogenic agents act to inhibit neovessel formation, measurements of preexisting microvessel density are not sufficient to reveal the functional or angiogenic status of the tumor neovasculature. Therefore, blood flow in the tumor, including the blood volume and the blood flow velocity in the microcirculation of tumor tissue, may be more sensitive than the blood vessel density for evaluating the efficiency of antiangiogenesis agents in the earlier stage of the drug administration. As a histological index, the blood vessel density could not be expected to change as quickly as the blood volume and velocity in the micro-circulation of the tumor in response to the treatments. Therefore MRI and ultrasound, as noninvasive modalities for determining the morphological and functional blood volume changes in the tissue are useful and valuable tools [15] for evaluation of the microvasculature during tumor growth, and also for monitoring as well as evaluating the functional characteristics of vasculature in the tumor as an indicator of the efficiency of anti-vascular and anti-angiogenic agents. 

One study recently demonstrated that tumor blood flow measured by destruction-replenishment ultrasound was highly correlated with the contrast enhancement estimated by CT images [30]. Our data indicated that relative blood volume and blood vessel density in the microcirculation of tissue measured by MRI is consistent with the vessel density determined by micro-CT. The results indicated also that the MRI measurements of rBVF and rBVD in the tumor reflect the histological structure in the tissue.

## 7. Study Limitations

One limitation of the rBVF and rBVD evaluation is that the magnetic resonance macromolecular agent, Clariscan, may slowly leak out of the blood vessels. All data was collected within 1.5 hours of contrast injection in this study; this should minimize the effects of leaking. Another problem is that we need blood samples for contrast agent concentration calculation in the blood pool, which is time consuming. One could measure the signal from a relatively large blood vessel *in vivo* instead in future studies. 

The relationship of the vascularity and volume of the tumor may be different when the volume of tumor is less than 2.0 cm^3^; however that was not included in this study. Micro-CT and high-frequency ultrasound may be helpful for evaluation of smaller tumors.

## 8. Conclusion

Relative blood volume in the microcirculation of tissue as measured by MRI is valuable for determination of the vascular, functional, and anatomic characteristics of a tumor, as well as the changes with growth. The observations using MRI and micro-CT have shown that rBVF and rBVD are significantly related to the characteristics and status of tumor growth. This has immediate utility for preclinical research studying the impact of novel therapeutics on tumor blood supply. Specifically, the blood volume determination in the tumor using the noninvasive MRI study may be useful for monitoring and evaluating the efficiency of anticancer drugs, especially anti-vascular and anti-angiogenic agents. Such studies may also help elucidate optimal timing in the administration of anti-vascular cancer therapies. This noninvasive MRI technique could be extended to clinical studies if and when an intravascular contrast agent similar to Clariscan is approved for patient use.
